# Mating First, Mating More: Biological Market Fluctuation in a Wild Prosimian

**DOI:** 10.1371/journal.pone.0004679

**Published:** 2009-03-05

**Authors:** Ivan Norscia, Daniela Antonacci, Elisabetta Palagi

**Affiliations:** Centro Interdipartimentale Museo di Storia Naturale e del Territorio, Università di Pisa, Calci (PI), Italy; Georgia State University, United States of America

## Abstract

In biology, economics, and politics, distributive power is the key for understanding asymmetrical relationships and it can be obtained by force (dominance) or trading (leverage). Whenever males cannot use force, they largely depend on females for breeding opportunities and the balance of power tilts in favour of females. Thus, males are expected not only to compete within their sex-class but also to exchange services with the opposite sex. Does this mating market, described for humans and apes, apply also to prosimians, the most ancestral primate group? To answer the question, we studied a scent-oriented and gregarious lemur, *Propithecus verreauxi* (sifaka), showing female dominance, promiscuous mating, and seasonal breeding. We collected 57 copulations involving 8 males and 4 females in the wild (Berenty Reserve, South Madagascar), and data (all occurrences) on grooming, aggressions, and marking behaviour. We performed the analyses via exact Spearman and matrix correlations. Male mating priority rank correlated with the frequency of male countermarking over female scents but not with the proportion of fights won by males over females. Thus, males competed in an olfactory tournament more than in an arena of aggressive encounters. The copulation frequency correlated neither with the proportion of fights won by males nor with the frequency of male countermarking on female scents. Male-to-female grooming correlated with female-to-male grooming only during premating. Instead, in the mating period male-to-female grooming correlated with the copulation frequency. In short, the biological market underwent seasonal fluctuations, since males bargained grooming for sex in the mating days and grooming for itself in the premating period. Top scent-releasers gained mating priority (they mated first) and top groomers ensured a higher number of renewed copulations (they mated more). In conclusion, males maximize their reproduction probability by adopting a double tactic and by following market fluctuations.

## Introduction

In biology, as well as in economics and politics, power is a key concept for understanding asymmetrical dyadic relationships [1]. Distributive power [2] can originate from both dominance (when force is used) and leverage (when the use of force is not possible). An individual has leverage over another when that individual possesses something that the other needs but cannot acquire through coercion [3]. In this case, trading becomes essential for mutually beneficial interactions within social groups, both in economical and biological markets [4]. An important feature of market models is that the expected future gains are actively influenced by playing off potential partners against each other [5], [6]. The typical game theory approach includes only two players and, although this is changing within economics as well as biology, the classical models do not take into account partner choice [4]. In contrast, the biological market theory includes multi-player models, that is theoretical games with at least three or more “players” (traders, in the market systems) [7]. Two or more classes of traders (sex classes, rank classes, etc.) exchange commodities in biological markets to their mutual benefit. Different group members can offer different kinds of commodities in exchange for alternative ones that they do not currently possess [4]. Usually, competition acts as the driving force within the same trader class (including all members offering the same kind of commodity) while cooperation can occur between different trader classes [4], [8].

In the mating market, the balance of power tilts in favour of females whenever males cannot force females into mating (as it happens in sexually monomorphic species or when females form coalitions) [3]. Consequently, males depend on females for breeding opportunities and must compete to prove their superiority to females, thus increasing their possibility to be selected [3], [9]. Males can engage in both contest competition via physical/ritualized fighting and outbidding competition, in which a male plays off rivals by making a better offer [4]. In the latter case, males can secure the favours of a female by advertising their quality (e.g. the dominance status) through visual or olfactory displays [10], [11] and/or by being more generous than others in providing a commodity in exchange for female access (competitive altruism) [8], [12]. One of the most valuable commodity that can be offered in social mammal groups is grooming, which is used for parasite removal [13], stress reduction [14], and as social cement to start, consolidate, or repair relationships [15]. Grooming is a commodity that can be exchanged for itself or for breeding opportunities [16].

**Figure 1:**
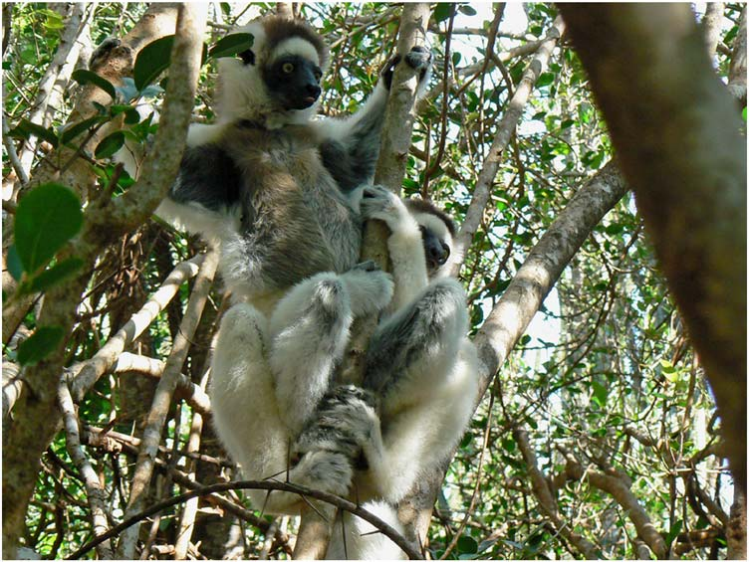
Sifaka copulation: picture taken during a mating episode. Photo by Daniela Antonacci *via* Panasonic Lumix DMC FZ7 - 12× optical zoom/36–432 mm equivalent/LEICA lens.

Sociality is widespread among mammals [17] and particularly among anthropoid primates (monkeys and apes [18]). In prosimians (the most ancestral group of primates) sociality is the exception more than the rule. Among Malagasy prosimians (lemurs), few species combine a powerful olfactory system (retained from basal mammals) and puzzling features like group living, female priority over resources, and absence of sexual dimorphism [19]. Such combination of features makes gregarious lemurs the ideal model to understand the biological bases of mate selection by females, who cannot be accessed by force or using food as exchange commodity. In particular, we selected the diurnal species *Propithecus verreauxi* of south/southwest Madagascar [20] to find out which male strategies are successful to maximize breeding opportunities (Figure 1).

## Results

The rank of mating priority assigned to males did not correlate with their mating frequency (Exact Spearman r_s_ = −0.434, n = 8, p = 0.284; α = 0.01 adjusted via Bonferroni).

Male priority rank correlated with the frequency of male countermarking (Exact Spearman r_s_ = 0.866, n = 8, p = 0.005; α = 0.01) but did not correlate with i) the proportion of fights won by males in presence of females (Exact Spearman r_s_ = 0.448, n = 8, p = 0.265; α = 0.01) and ii) the frequency of grooming directed by males to females (Exact Spearman, r_s_ = −0.099, n = 8, p = 0.816; α = 0.01) and by females to males (Exact Spearman, r_s_ = 0.138, n = 8, p = 0.744; α = 0.01).

The mating frequency correlated neither with the proportion of fights won by males in presence of females (Kr = 22, τ_Kr_ = 0.284, P = 0.057, α = 0.0125 adjusted via Bonferroni) nor with the frequency of male countermarking on female depositions (Kr = 16, τ_Kr_ = 0.209, P = 0.103). In the breeding period, mating frequency correlated with the frequency of grooming directed by males to females (MF grooming; Kr = 26, τ_Kr_ = 0.609, P = 0.001, α = 0.0125 adjusted via Bonferroni) but not with the frequency of grooming performed by females to males (FM grooming; Kr = 12, τ_Kr_ = 0.336, P = 0.091).

**Figure 2:**
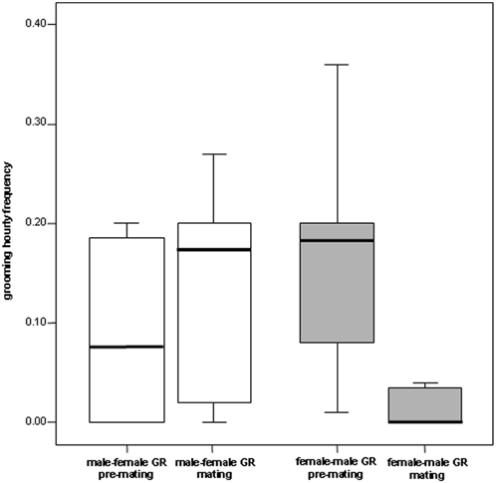
Grooming interchange. Variation in the levels of grooming directed from males to females and from females to males in the two periods (premating and mating).

MF grooming and FM grooming correlated in the premating period (Kr = 28, τ_Kr_ = 0.675, P<0.001, α = 0.0125 adjusted via Bonferroni) but not in the mating days (Kr = 3, τ_Kr_ = 0.157, P = 0.282). FM grooming significantly decreased in the mating days compared to the premating period (Wilcoxon Signed Ranks Test T = 0, P = 0.008, n = 8) while MF grooming did not differ between the two periods (Wilcoxon Signed Ranks Test T = 6, P = 0.102, n = 8) (Figure 2).

## Discussion

Whenever individuals cannot forcibly appropriate valuable resources without the consent of the owner, they should compete for partners and negotiate about resource distribution in biological markets [4]. Specifically, the mating market should involve a) male-male competition to gain female access and b) male-female trade, in which males bargain services for breeding opportunities [21], [22], [23].

### Male-male competition for female access

Aggressive interactions are a widespread form of competitive strategy adopted by males to gain female access [24]. However, in the sifaka we found no correlation between the proportion of fights won by each male and mating frequency and priority. Since the sifaka society is characterized by female dominance and philopatry [25], it is not surprising that male fighting ability is unimportant in female mate choice. In general, winning a fight does not necessarily confer sexual access on males. In fact, females can base their mate choice on other features (e.g., age, time spent in the group, male physiological status, etc.) especially in those species in which females can acquire a dominant or co-dominant status, individually or by forming coalitions [26], [27], [28].

In scent oriented species, male competition for females can be translated into an olfactory tournament (outbidding competition) more than into an arena of aggressive encounters (contest competition) [9], [11], [29], [30], [31]. Scent marks provide a reliable signal of competitive ability [29], [28], [32]. Sifaka males competed for females by countermarking female odour depositions: in the end, the most active males gained breeding priority (Video S1). As a matter of fact, sifaka males can use scent marking as a form of self-advertisement for mating purposes [33] possibly because odour signals convey information on dominance status, which is one of the main choice criteria adopted by females [24], [34]. The importance of olfactory male competition in female mate choice has been provided for non primate species [29] as well as for primate ones, including New World monkeys [35] and prosimians. In particular, females of *Nycticebus pygmaeus* (a nocturnal prosimian) rely on olfactory deposition frequency to select mating partners [36]. Moreover, during the premating period *Lemur catta* males compete for female access via ritualized “stink fights” and females increase their tolerance towards males based on the outcome of such fights [37], [38].

### Male-female *do ut des* for breeding opportunities

In the sifaka, the higher mating priority gained by males via scent marking activity did not match with a higher number of copulations. In fact, mating first does not necessarily mean mating more. In order to increase their breeding opportunities, males had to move from theory to facts, by offering a service in exchange for sex (mating market) after a self-promotion phase via odour messages.

Grooming and food are the two main valuable commodities that a male can offer to a female [24], [39], [40], [41], [42]. However, food is not a spendable commodity in the sifaka society, since females have unquestioned feeding priority [43]. We found that in the premating period grooming performed by males to females positively correlated with grooming performed by females to males (grooming reciprocity). Instead, in the mating period we found that grooming performed by males to females was correlated with the frequency of copulations but not with grooming received by males from females (Video S2). These results indicate that grooming was traded for itself in the premating period (interchange) and for mating opportunities in the mating period (exchange). In short, males used the same commodity across the study period, whereas females switched from grooming to breeding availability during the mating period.

Grooming market has been found also in other primate species. Barrett and Henzi [39] found that in chacma baboons (*Papio cynocephalus ursinus*) grooming exchanged within females was affected by the rank distance between individuals. Similarly, Port et al. [44] found that in the redfronted lemur (*Eulemur fulvus rufus*) grooming trade was influenced by rank position. In fact, subordinates traded grooming for itself with other subordinates and for social tolerance with dominants [44]. The fact that sifaka females can mate also with out-group individuals [45] indicates that mate choice by females goes beyond the relative ranking status within males belonging to a stable foraging group. Yet, by chest condition (stained; Palagi et al., unpublished data), we can infer that out-group males were probably high ranking individuals in their groups of origin.

The exchange of grooming for sexual access is not uncommon even in societies characterized by male dominance [39], [46]. In fact, regardless of the dominant sex, the leverage of females increases when they are in oestrous because they have an inalienable commodity: their eggs ready to be fertilized [3]. For example, male baboons use grooming to ensure females tolerate them in close proximity so that they can exclude other males and achieve a high frequency of mating [39], [47]. In chimpanzees, low ranking males need to provide more grooming to oestrus females than high ranking males in order to gain female access [42]. Beyond primates, Stopka and MacDonald [40] found that females of *Apodemus sylvaticus* (a mouse species characterized by promiscuous mating system without any paternal investment) require grooming before allowing a male to progress towards sex. The same authors hypothesized that females could obtain grooming through a process of “unintentional bargaining” [*sensu* 40]: in such species, grooming was the only commodity which males had been seen to provide in the process of mate selection.

In conclusion, mate choice by sifaka females is complex and a single factor cannot explain it all. Many males can compete and occasionally obtain female access but only top scent-releasers and groomers reach the highest mating priority and rates, thus maximizing their reproduction probability. On a broader perspective, we demonstrated not only that the biological market paradigm can successfully be applied to prosimians but also that such market undergoes seasonal fluctuations, shifting from a grooming to a mating market over time.

## Materials and Methods

### Study species and site

We conducted this study in the secondary forest of Ankoba, in the Berenty Reserve (South Madagascar; S 24.99°; E 46.29°; for an extensive description see [48]) on *Propithecus verreauxi* (Verreaux' sifaka).

The sifaka are social and diurnal prosimians that live in relatively stable groups (spanning 2–13 individuals, e.g. at Beza-Mahafaly, South West Madagascar [49] and Kirindy, West Madagascar [33]). At Berenty, sifaka groups range from 1 to 10 individuals, according to a complete census conducted in November-December 2006 [50]. As with other lemur species, sifaka groups are characterized by an unbalanced sex-ratio, which is skewed towards males [19], [50], [51] (Table 1).

**Table 1:** Descriptive statistics of the sifaka counted and sexed at Berenty in 2006: total number of groups and individuals, number of adult males and females, and number of infants; minimum, maximum and standard deviation (STD) of the number of individuals (of both sexes), males, females, and infants per group [50].

	Total number	Min/Group	Max/Group	Mean	STD
Groups	49	1	10	4.22	2.16
All animals (infants and adults)	229	1	10	4.67	2.40
Adult Males	127	0	7	2.59	1.62
Adult Females	79	0	4	1.61	0.89
Infants	23	0	2	0.47	0.62

They inhabit riverine and dry forests of south and southwest Madagascar [20] and are sexually monomorphic (or females are larger than males; [52]). Moreover, the sifaka are characterized by female philopatry and social dominance and by the absence of male infant care [20]. Sifaka males are very active in scent marking via both sternal glands (abortive in females) and ano-genital secretions [37]. Moreover, sifaka males are bimorphic in chest status: the ones that are most active in scent marking show a pronounced brown staining around their sternal gland (stained chested males) while the others do not (clean chested males) [34]. Stained-chested males (different from clean-chested males) usually occupy a dominant position in sifaka groups [34]. Females usually experience a single oestrus period (2–3 days) per year and both sexes can mate with multiple partners in their own and neighbouring groups, especially when a single group offers suboptimal mating opportunities [53]. In particular, males can start roaming and visiting other groups in search of oestrus females [45]. The short oestrus period and the fact that mating can be tightly synchronized within a population make copulations very difficult to detect and observe [25], [34]. Moreover, at Berenty, cyclones and heavy raining followed by river flooding normally prevent data collection in the period January-February, coinciding with sifaka's mating period. In 2007, for the first time it was possible to gather data on mating because of a prolonged drought involving South Madagascar. In the end, we gathered the highest sample of mating episodes ever recorded in prosimians.

### Observational data and operational definitions

Mating, observed in one group, involved in-group members (6 males and 4 females) and 2 out-group males both showing a stained chest (all animals were individually identified according to their external features, [37]). Group composition and sex-ratio were typical for the study species in general [49] and for the study population in particular [50] (cf. Table 1). As reported at Beza-Mahafaly [45], also at Berenty males started visiting neighbouring groups prior to the mating days. As a matter of fact, several out-group males started visiting our study group 23 days before the first mating day. We were able to collect standardized data on two of them, which visited and spent 70% of time with the study group. It was not possible to pool out-group with resident males to draw a dominance hierarchy because the time spent by out-group males with residents was not enough to allow any statistical analysis in this respect.

The premating period was defined as the month prior to the mating days. The authors and a field assistant collected mating, grooming, aggressive interactions, and scent marks via all-occurrences (221 hr; [54]), during daily continuous observations (about 11 h/day) on both in-group and out-group members. Data were collected from December (2006) to February (2007) when the observations had to be stopped because of storming weather.

We collected 53 male-male aggressions, 551 male marking bouts, and 72 allo-grooming bouts. As typical of the sifaka the individuals of the group usually moved, rested, and foraged cohesively. However, the group could split during the mating days: in this case, the observers separated to follow the two different subgroups.

Brockman, who observed sifaka mating in a different study site (Beza-Mahafaly; Southeastern Madagascar; [22]), provided the operational definitions used during this study. In particular, mating referred to copulatory behaviour in which intromission and thrusting were unambiguously observed (Figure S1 and Video S3). During our study, copulations lasted from 11 sec to 7 min (N = 57, mean: 1.860 min±1.603 SE). Mount occurred for less than 3 sec without intromission and thrusting, and were usually associated with female resistance. Ejaculation, generally not visible, was inferred based on a rapid increase in thrusts and a pause just prior to the dismount, followed by intense genital self-grooming [45], [55]. In this study, only proper copulations were included in the analysis.

To calculate the mating priority index we first ranked males according to the order by which they accessed each oestrus female (male priority rank). When a male did not access to one oestrus female at all, the rank assigned to the male for that female was 0. Then, the rank sum for each male was averaged on the number of oestrus females. The male priority rank has not to be confounded with the hierarchical position of males within their own groups (dominance ranking position).

### Statistical analyses

The analyses were conducted at dyadic and individual level (N_males_ = 8; N_females_ = 4). Behavioural bouts per individual (mating episodes, aggressions, grooming, and scent marks) were normalized on the observation time (hours).

We used the Rowise Matrix Correlation test using rectangular matrices (MatrixTester 2.2.2b by Hemelrijk 2001) to verify the relationship between mating frequency and a) the proportion of fights won by males in presence of females, b) male countermarking on female depositions c) male-to-female and female-to-male grooming. With the same method we also tested the correlation between female-to-male and male-to-female grooming during the mating and premating days.

Due to the small sample size and deviation from normality (Kolmogorov-Smirnov<0.05) we used non parametric statistics (software: Statxact 8, Cytel Studio, and SPSS 12.0). In particular we adopted the Spearman test to correlate the rank of mating priority with the frequency of a) mating episodes; b) male countermarking on female depositions; c) fights won by males in presence of females; d) male-to-female and female-to-male grooming. Moreover we used the Wilcoxon match-pairs signed rank test to compare the frequency of male-to-female and female-to-male grooming between premating and mating days.

Exact values were applied following [56] and, when needed, the significance level (α = 0.05) was adjusted downward following the Bonferroni technique [57].

## Supporting Information

Figure S1 Details of a copulation (photo by Daniela Antonacci via Panasonic Lumix DMC FZ7 - 12× optical zoom/36–432 mm equivalent/Leica Lens)
(3.21 MB TIF)

Video S1 Male countermarking behaviour on a female scent deposition (video by Daniela Antonacci via Canon DM MV 600-18× optical zoom/2.8–50 mm equivalent/Canon Video Lens).
(10.01 MB MOV)

Video S2 Copulation followed by a grooming session (video by Daniela Antonacci via Canon DM MV 600-18× optical zoom/2.8–50 mm equivalent/Canon Video Lens).
(10.21 MB MOV)

Video S3 Copulation in which intromission and thrusting were unambiguously observed (video by Daniela Antonacci via Canon DM MV 600-18× optical zoom/2.8–50 mm equivalent/Canon Video Lens).
(9.99 MB MOV)
