# Supplementary figures and images for: Mating First, Mating More: Biological Market Fluctuation in a Wild Prosimian
Source: PLoS One. 2009 Mar 5;4(3):e4679. doi: 10.1371/journal.pone.0004679 (PMC2650411; doi:10.1371/journal.pone.0004679)

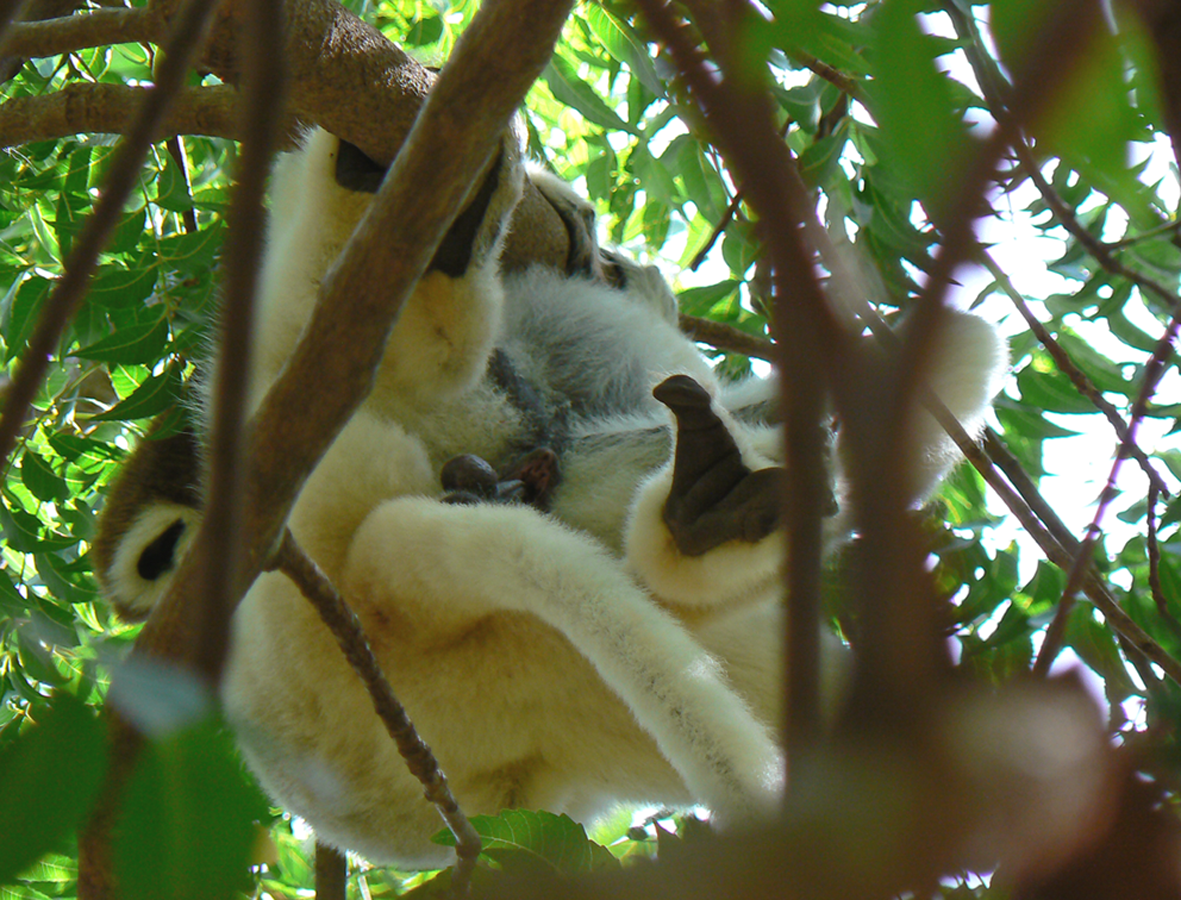

Supplement: Figure S1 — Details of a copulation (photo by Daniela Antonacci via Panasonic Lumix DMC FZ7 - 12× optical zoom/36–432 mm equivalent/Leica Lens) (3.21 MB TIF) [file pone.0004679.s001.tif]
